# Gateway to multifaceted food science and nutrition challenges

**DOI:** 10.1002/fsn3.3

**Published:** 2013-01-08

**Authors:** Y Martin Lo


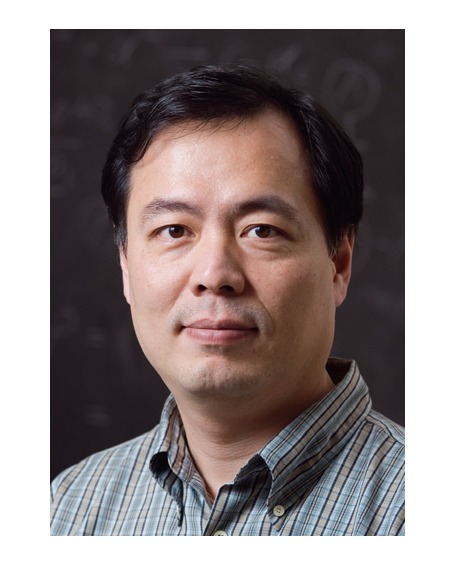


The science of food has evolved significantly over the last two decades with numerous specialized journals available to promote scientific communication. While most journals continue to push for cutting-edge technologies in order to boost their impact factor, it is important to point out that the full impact of research conducted in food science and nutrition cannot simply be represented by a count of short-term citations. It has become clear that integrated research to interpret the interface between food science and nutrition is critical to attaining a full understanding of functional foods. Additionally, many countries are still in dire need of appropriate technologies to add value to their abundant agriculture products and byproducts, while developed countries are seeking better, more sustainable agricultural practices. Such efforts are time intensive and need a proper outlet to be shared with as wide an audience as possible; we therefore proudly introduce a new open access journal to promote continuous development and stimulate rapid, effective communication. It is my vision for *Food Science & Nutrition* to be the all-inclusive, multidisciplinary hub for these integrated efforts.

I am delighted to welcome you to *Food Science & Nutrition*, a new open access, multidisciplinary journal that aims to publish high-quality and high-impact original research on food science and nutrition. Interwoven by the globalization of our food supplies, food safety and security enters a new era with many intrinsic challenges and opportunities. Globalization concurrently rises with the “buying-local-eating-local” trend of today's organic-seeking consumers, leading to increasingly complex factors to be studied. In both global and local arenas, the interface between food science and nutrition inevitably starts to take precedence over traditional, single-field research. Plenty has been published about functional ingredients and nutraceuticals, but not enough about the bioavailability of such ingredients. For instance, in vitro bench-top analytic protocols do not fully address the issue that the same phytochemical recovered from different plants may have different bioavailability. More bio-based assays would need to be developed to gauge the effectiveness of each of the functional ingredients, and these studies should be published. Characterization of the biocompatibility of the wide variety of nutraceuticals is another area of research that should be thoroughly investigated and represented in the literature.

Furthermore, there is an increasing need for vertical integration of food science and nutrition from farm to fork and beyond. Food safety control of fresh-cut produce needs to begin on the farm with effective strategies for pathogen control in soil and surrounding environment throughout the processing and distribution chain in order to minimize food-borne outbreaks. System-wide assessment is needed to identify food safety risks for effective interventions. Rapid detection of food-borne pathogens needs to work with a broad range of food matrices, now packaged in increasingly versatile materials.

Frontline detection of adulterations is of significant importance, as outbreaks resulting from food-borne pathogens in one part of the world understandably lead to worries in the global marketplace. These challenges have inherent political implications, and relevant research advancements in the areas of food science and nutrition are worthy of open-minded, academic discussion. We must gather more direct evidence before we can make wise recommendations for food safety regulations that can effectively protect today's global food supplies.

I am excited to present our field with the opportunity to collaboratively tackle these global issues by way of publishing in an open access platform, providing global access to the most important research findings in food science and nutrition. *Food Science & Nutrition* is committed to speed and innovation in research communication and to maximizing the visibility of each published article. Furthermore, because it is an open access, online-only journal without article limits or page restrictions, *Food Science & Nutrition* can publish more articles in emerging, interdisciplinary research areas, as well as confirmatory studies that are critical to the literature, but often overlooked by other journals where space is at a premium. This also allows authors more space to respond to the comments of reviewers and to incorporate some of the valuable debate that often occurs between authors and reviewers behind the scenes into the article itself for the benefit of all readers.

I look forward to receiving contributions from leading scientists as well as rising academic stars to share their viewpoints and scholarly contributions of fundamental and applied research related to all aspects of human food and nutrition, as well as interdisciplinary research that spans these two fields. Applicable topics include, but are not limited to, the following areas:

- Chemistry of food and its biochemical interactions
- Food microbiology, safety, and risk assessment
- Metabolic, molecular, and genetic mechanisms in nutrition
- Safety and security analysis of global food supplies
- Food preservation, storage, and hurdle technology
- Food toxicology
- Engineering of food processing technologies
- Handling and packaging of foods
- Quality assurance of food products
- Biotechnology as it relates to food production and processing
- Food oral processing, rheology, and other texture-related studies
- Health and nutritional implications of food, functional foods, nutraceuticals, and supplements
- Bioavailability and disease prevention
- Nutritional methodologies, behaviors, and modeling
- Sensory and consumer science
- Community and international nutrition
- Enology and fermentation technology
- Food and dietary supplement ingredient regulatory science
- Health claims
- Agriculture research on plant production, utilization, biomass, and environment
- Commentaries on controversial issues in food science and nutrition
- Interdisciplinary research spanning food science and nutrition

*Food Science & Nutrition* welcomes the submission of original research articles, systematic reviews, meta-analyses, and research methods papers, along with invited editorials and commentaries. *Food Science & Nutrition* also works in partnership with other journals published by Wiley to give authors of high-quality work, which cannot be accommodated in these leading journals, the opportunity to transfer their manuscript for consideration by *Food Science & Nutrition*. Together with our publisher, our international editorial board of experts and I aim to create a truly global forum for high-quality research and to make this important work widely accessible as quickly as possible. It is our belief that this journal will serve as an excellent platform for integrated research, education, and outreach of food science and nutrition serving a global community of researchers, authors, and readers.

